# Potential Large-Scale CO_2_ Utilisation for Salicylic Acid Production via a Suspension-Based Kolbe–Schmitt Reaction in Toluene

**DOI:** 10.3390/molecules29112527

**Published:** 2024-05-27

**Authors:** Omar Mohammad, Jude A. Onwudili, Qingchun Yuan

**Affiliations:** Energy and Bioproducts Research Institute, College of Engineering and Physical Sciences, Aston University, Birmingham B4 7ET, UK; mohammao@aston.ac.uk (O.M.); q.yuan@aston.ac.uk (Q.Y.)

**Keywords:** suspension-based carboxylation, phenolics, Kolbe–Schmitt, hydroxy aromatic carboxylic acids (HACAs), CO_2_ utilisation, Net Zero

## Abstract

Conversion of CO_2_ into organic chemicals offers a promising route for advancing the circularity of carbon capture, utilisation, and storage in line with the international 2050 Net Zero agenda. The widely known commercialised chemical fixation of CO_2_ into organic chemicals is the century-old Kolbe–Schmitt reaction, which carboxylates phenol (via sodium phenoxide) into salicylic acid. The carboxylation reaction is normally carried out between the gas–solid phases in a batch reactor. The mass and heat transfer limitations of such systems require rather long reaction times and a high pressure of CO_2_ and are often characterised by the low formation of undesirable side products. To address these drawbacks, a novel suspension-based carboxylation method has been designed and carried out in this present study, where sodium phenoxide is dispersed in toluene to react with CO_2_. Importantly, the addition of phenol played a critical role in promoting the stoichiometric conversion of phenoxide to salicylic acid. Under the optimal conditions of a phenol/phenoxide molar ratio of 2:1 in toluene, a reaction temperature of 225 °C, a CO_2_ pressure of 30 bar, a reaction time of 2 h, and stirring at 1000 rpm, an impressive salicylic acid molar yield of 92.68% has been achieved. The reaction mechanism behind this has been discussed. This development provides us with the potential to achieve a carboxylation reaction of phenoxide with CO_2_ more effectively in a continuous reactor. It can also facilitate the large-scale fixing of CO_2_ into hydroxy aromatic carboxylic acids, which can be used as green organic chemical feedstocks for making various products, including long-lived polymeric materials.

## 1. Introduction

Global CO_2_ emissions from the use of fossil fuels was 37.55 billion tonnes in 2023 and is expected to increase in the coming years due to increased energy demand [1]. Through these activities, CO_2_ concentrations in the atmosphere has surpassed an unprecedented value of 410 ppm [2], leading to global warming, climate change, and their deleterious consequences. Hence, there is a need to reduce CO_2_ emissions to avoid the predicted environmental consequences of increased global temperatures. The most obvious way to reduce the concentration of CO_2_ in the atmosphere is to halt its release from burning carbon-based fuels, particularly those derived from fossil resources. Another effective approach involves industrial-scale carbon capture, utilisation, and storage (CCUS) technologies. Currently, around 230 million tonnes of CO_2_ are used annually as raw materials and ingredients across various sectors [3]. For chemical fixation to significantly impact CO_2_ emission reduction, its application in producing durable chemical products must be promoted.

The chemical fixation processes being developed include producing inorganic materials like limestone and aggregates, and organic compounds like methanol, cyclic carbonates, hydroxy aromatic carboxylic acids (HACAs), and polycarbonates [4,5,6,7,8]. Although the current demand for CO_2_-based organic chemicals is insufficient to substantially reduce emissions, this approach remains vital within a comprehensive “all technologies” strategy [3,9]. For instance, producing 100 million tonnes of methanol annually requires around 140 million tonnes of CO_2_, representing only 0.4% of current emissions [10]. Future demand for methanol and other CO_2_-based chemicals may increase, making this approach more promising. However, using CO_2_-derived methanol as fuel only allows short-term CO_2_ storage.

In contrast, HACAs have significant potential due to their versatile applications including in pharmaceuticals [11,12,13], dyes [14,15], fragrances [16], cosmetics [17,18], food supplements [18,19], and biodegradable plastics [20,21,22]. Increasing the production and the use of durable materials from HACAs offers long-term carbon storage, essential for the CCUS strategy. 

**Scheme 1:**
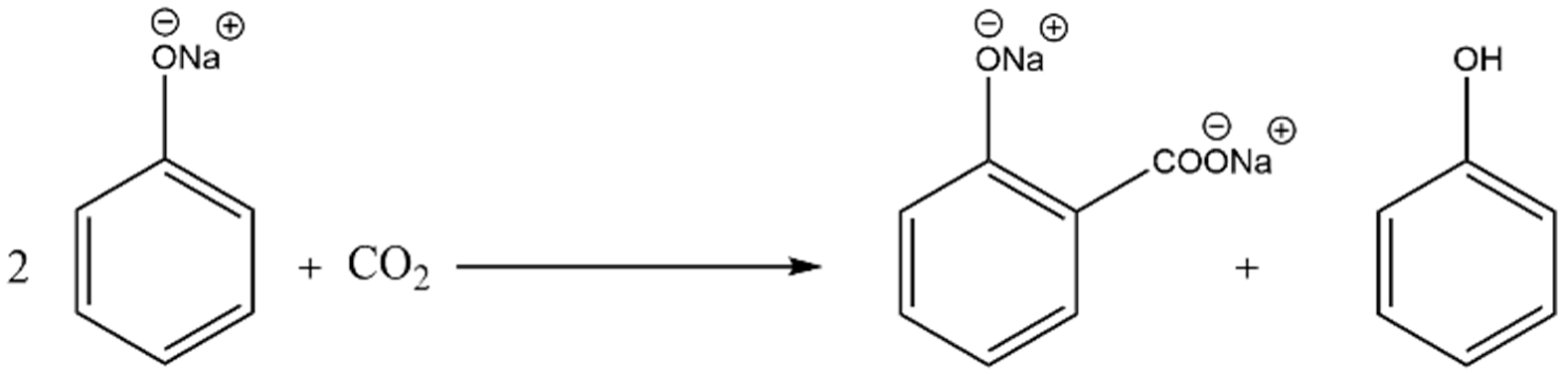
The stoichiometric equation suggested by Kolbe. Two moles of sodium phenoxide react with one mole of CO_2_ forming one mole of salicylic acid salt and one mole of phenol.

Among HACAs, salicylic acid is particularly of huge commercial interest [23]. Recent research suggests that salicylic acid and acetic acid could develop recyclable vinyl polymers, reducing reliance on polymer vinyl chloride (PVC), which has an annual global production of 44.2 million tonnes [24]. Despite its importance, salicylic acid is still produced using the conventional Kolbe–Schmitt reaction, initially invented by Kolbe, which involved heating a mixture of phenol and sodium in the presence of CO_2_ [25]. Kolbe’s subsequent experiments with sodium phenoxide as the starting material demonstrated higher yields of salicylic acid. However, the volatilisation of phenols restricted the yield to less than 50% of the stoichiometric amount. Kolbe indicated that for every two moles of sodium phenoxide heated in a stream of CO_2_, almost exactly one mole of phenol was liberated (Scheme 1).

Recognising these challenges, Schmitt made modifications by conducting the reaction with dry sodium phenoxide and CO_2_ in a closed vessel under high pressure and temperature conditions [7]. This adjustment prevented the volatilisation of phenols and improved mass transfer, leading to significantly higher yields of salicylic acid. The Kolbe–Schmitt reaction yielded up to 79% salicylic acid after 8 h of reaction at 125 °C and CO_2_ pressures ranging from 82 to 138 bar [26]. These stringent conditions facilitated the conversion of released phenol back to phenoxide for subsequent salicylate formation, overcoming kinetic and equilibrium barriers, becoming more thermodynamically favoured. Consequently, the modified Kolbe–Schmitt reaction has become the standard industrial procedure for preparing a wide range of HACAs [27].

However, challenges persist due to extended reaction times, high pressures, and inadequate mass/heat transfer inherent in the batch mode of the gas–solid reaction. These issues often lead to inefficient mixing, local hotspots, and enhanced formation of undesirable side products, thus hindering the maximisation of expected yields. Therefore, much efforts have been focused on developing other carboxylation methodologies, such as the use of novel activation/carboxylation agents (e.g., ionic liquids) [28,29,30], heterogeneous catalysts (e.g., carbon-coated Fe-Al_2_O_3_) [31,32,33] and homogeneous stoichiometric reagents (e.g., 1,8-diazabicyclo[5.4.0] undec-7-ene (DBU)) [34,35,36,37]. Yet, none of the developed methods have been applied at an industrial scale due to their limitations such as high production costs and low yields. More details and industrial limitations of those methods can be found in a recent review paper [23].

Other research efforts have aimed to address the limitations in mass/heat transfer between gas and solid reactants by suspending sodium phenoxide in solvents. Various solvents, including alcohols (methanol, ethanol, and 1-butanol), glycol, glycerol, aromatic xylene, and di-isobutyl ketone, have been utilised to modify the traditional Kolbe–Schmitt reaction [27,38]. Table 1 summarises that solvents with high dielectric constants inhibit salicylic acid formation, whereas those with low dielectric constants facilitate the desired reaction. Salicylic acid yield exhibits an increasing trend with decreasing dielectric constant. Notably, xylene demonstrates the highest salicylic acid yield of 33.5%, with a dielectric constant of 2.6 at 20 °C. These findings suggest that solvents with low dielectric constants and low solubility are advantageous for suspension-based routes.

**Scheme 2:**
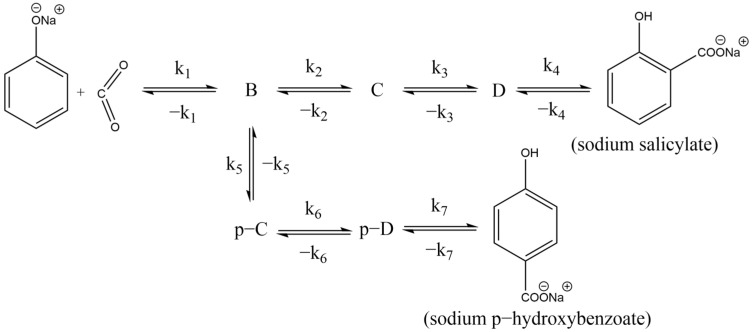
Series of consecutive and simultaneous reaction in the Kolbe−Schmitt mechanism, where B, C and D are the proposed intermediates by Markovic et al. [39].

These findings were consolidated by a theoretical study using density functional theory (DFT), when it was suggested that the reaction proceeded via the four step-reaction mechanism model of Markovic [39]. However, the reaction steps became reversible in the presence of solvent (Scheme 2), including the fourth irreversible step in the solvent-free reaction conditions [40]. Although the reaction rates increased in the presence of a solvent, the reversibility of the fourth step (k_4_) could result in little or no formation of the desired product, depending on its solubility in the solvent. Since then, no study has focused on improving the suspension-based carboxylation reaction. Due to its thermodynamic limitation in the presence of a solvent, studies on improving the suspension-based carboxylation reaction appear to be scarce in the literature.

For instance, the first continuous carboxylation was successfully achieved using dihydric-phenol (resorcinol) with aqueous KHCO_3_, increasing space–time yield by a factor of 440 compared to batch processing [35]. However, this method did not work for mono-hydric-phenols due to difficulties of inducing enough nucleophilicity to attack the carbon atom of CO_2_.

**Figure 1:**
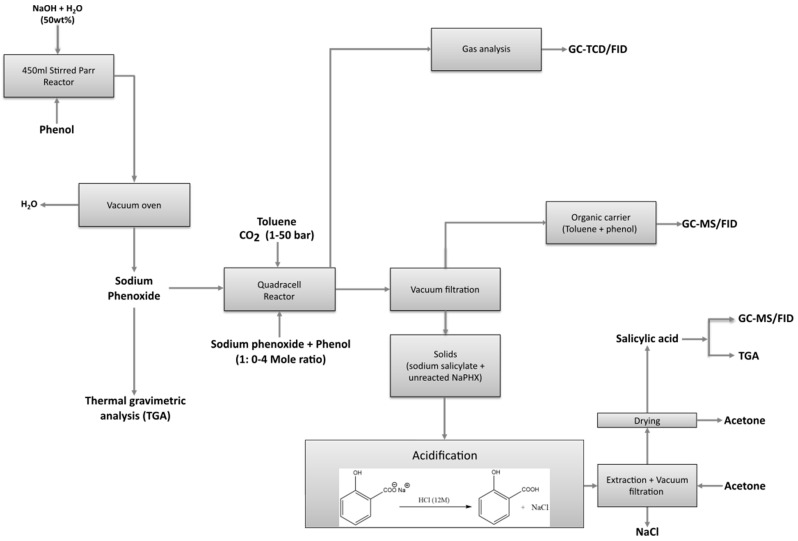
A schematic summary of the methodology of the novel suspension-based carboxylation reaction.

Taking these experimental and theoretical barriers into account, toluene was selected as a solvent due to its dual advantage of ability to dissolve CO_2_ and low dielectric constant [41,42], to develop an innovative suspension-based Kolbe–Schmitt reaction. If successful, this would provide important and novel research data for potential development of a continuous process for effective large-scale fixing of CO_2_ into stable organic chemicals. In this present study, phenol was first converted into sodium phenoxide, which was then reacted with CO_2_ in toluene solvent as reaction medium, with the aim of producing salicylic acid. The study investigated the effects of temperature, reaction time, CO_2_ pressure, stirring speed and additional phenol as promoter on salicylic acid yield and purity during this suspension-based Kolbe–Schmitt-type carboxylation to obtain data for potential optimisation. The overall experimental method is depicted in Figure 1 and fully described in Section 3.

## 2. Results and Discussion

### 2.1. Confirmation of Prepared Sodium Phenoxide by TGA

**Figure 2:**
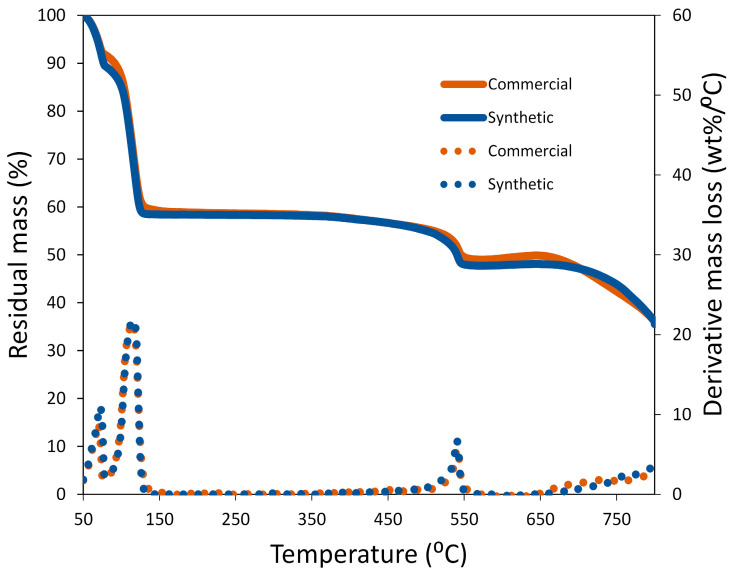
TGA thermograms for the mass loss and derivative mass loss of commercial and synthesised sodium phenoxide samples.

**Figure A1:**
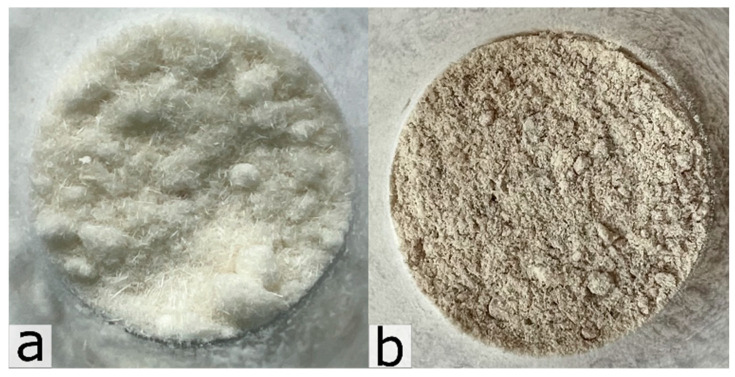
Photos of sodium phenoxide samples. (**a**) Sodium phenoxide obtained commercially. (**b**) Sodium phenoxide synthesised in the lab (with no further purification).

A commercial sodium phenoxide sample was procured for comparison with the prepared sample (see Figure A1 in Appendix A for photos of the two samples). The thermogravimetric analyses of commercial sodium phenoxide and the synthesised sample (according to Kolbe [25,27]) were conducted to observe their thermal decomposition patterns in relation to increasing temperatures. The generated TGA thermograms (Figure 2) for both commercial and prepared sodium phenoxides follow identical weight loss (wt%) with respect to temperature, as well as the derivative mass loss (wt%/°C).

The weight loss observed between 50 and 110 °C was attributed to the loss of water. It should be noted here that the amount of water indicated in the TGA spectra count have surpassed the actual water contents of the samples due to the hygroscopic nature of the sodium phenoxide [25] and the unavoidable exposure of solids to air in the TGA autosampler. Further derivative mass loss (wt%/°C) peak was observed at around 550 °C. This observation aligns with a recent study, which revealed that the initial step of thermal degradation of sodium phenoxide occurred at 550 °C, resulting in the formation of detectable benzene as the sole gaseous species in the TGA-FTIR spectra [43]. The thermal degradation behaviour of sodium phenoxide differs from that of phenol due to the substitution of -OH by -Ona, which reduces the thermal stability of the phenoxide ion [43]. The tautomerization to the keto form, followed by the loss of CO and the concurrent formation of cyclopentadiene, contributes to the reduced thermal stability of sodium phenoxide. This degradation process is typically initiated at 650 °C [43], as supported by the TGA thermogram in Figure 2, with evidence of a substantial mass loss around this temperature.

### 2.2. Effect of Reaction Parameters on the Yields of Salicylic Acid and Phenol

Preliminary experiments were initially carried out to establish the possible stoichiometry of the reaction between sodium phenoxide and CO_2_ [41]. The results from these tests were compared with Kolbe’s report on the formation of phenol during carboxylation. These analyses revealed that for every two moles of sodium phenoxide used, the reaction yielded one mole each of salicylic acid and phenol. The phenol produced from the Kolbe–Schmitt reaction is denoted as ‘reaction phenol’ in this present work to distinguish it from any added phenol, where applicable. The remarkable realisation of this stoichiometric molar balance not only provided crucial insights into the mechanisms of the reaction but also provided a confirmation to the hypothesis of the Kolbe reaction. This formed the basis for subsequent parametric experimental programme used in this present study. In all experiments, the yields of products have been reported in mole percent.

#### 2.2.1. Effect of Toluene Solvent and Phenol Addition

Toluene was selected as the dispersion solvent due to its wide industrial availability, low dielectric constant and the ability to dissolve CO_2_ [41,42]. Under reaction conditions of 150 °C, CO_2_ pressure of 30 bar and a reaction time of 2 h, the reaction of sodium phenoxide generated a salicylic acid yield of 18.70%. Notably, the literature reported low salicylic acid yields (Table 1) due to inhibition of the Kolbe–Schmitt reaction in the presence of a solvent [27,40].

**Figure 3:**
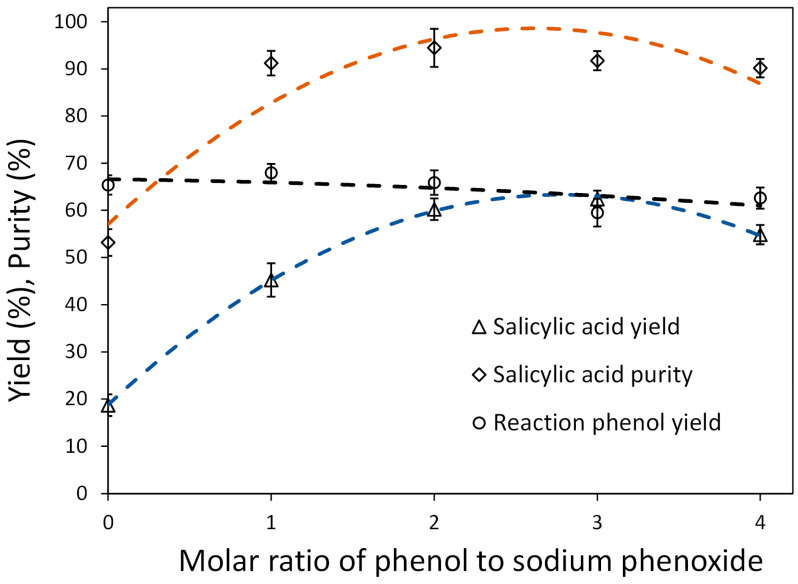
Effect of phenol on the yield and purity of salicylic acid during the slurry-based carboxylation reaction. The reaction conditions are as follows: reaction temperature 150 °C, reaction time of 2 h, Pco_2_ = 30 bar, and stirring at 1000 rpm.

The influence of phenol addition on the yield of salicylic acid, experiments were carried out with phenol/sodium phenoxide molar ratios of 1:1, 2:1, 3:1 and 4:1, respectively, while keeping other experimental variables constant (temperature of 150 °C, CO_2_ pressure of 30 bar and stirring speed of 1000 revolutions per minute (rpm)). The results of these tests are presented in Figure 3 in terms of salicylic acids yields (mole %). Compared to the experiment without added phenol, the yields of salicylic acid increased to 45.23%, 60.21%, 62.39% and 54.82%, respectively, with corresponding increase in phenol concentrations. The highest salicylic acid yield was obtained at a molar ratio of 3:1, which was a marginal increase compared to the 60.21% yield at a ratio of 2:1. When the concentration of phenol was further increased to reach a molar ratio of 4:1, the yield of salicylic acid dropped to 54.82%. The purity of the salicylic acid product followed the same pattern as the measured yields in the presence of increasing amounts of added phenol (please see the definition of purity in the Materials and Methods Section 3.8).

**Scheme 3:**
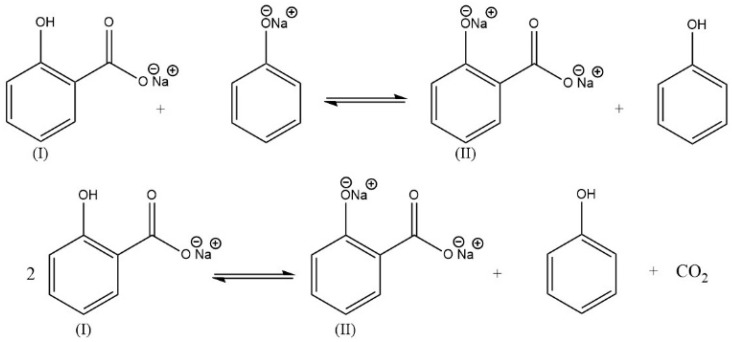
Side reactions proposed by Kolbe–Schmitt [27].

The results could be explained in line with the reactions shown in Scheme 3, which are the reversible side reactions in the Kolbe–Schmitt mechanism [27]. Without phenol addition, the desired (mono)sodium salicylate (I) would tend to react with itself or sodium phenoxide to form disodium salicylate (II) and phenol. However, with the addition of phenol, the equilibrium of the reversible reactions would be shifted towards the left-hand side, limiting further reactions of the formed monosodium salicylate (I) into disodium salicylate (II). The shift in equilibrium could also prevent the undesired consumption of the reactant sodium phenoxide. Consequently, the addition of phenol seemed to have increased the yield of monosodium salicylate (I).

To verify the proposed side reactions of the Kolbe–Schmitt reaction in Scheme 3 [27], sodium salicylate (I) was obtained through the carboxylation of sodium phenoxide and CO_2_ (dry-basis). Subsequently, sodium salicylate (I) was suspended in toluene, heated to 150 °C and held for 2 h. The results revealed a 31.4% molar yield of phenol and a 33.8% molar yield of CO_2_, providing evidence of the reversibility of the bottom reaction in Scheme 3 in a pure toluene solvent. Hence, the monosodium salicylate (I) reacted with itself to produce disodium salicylate (II), CO_2_ and phenol. Therefore, the presence of added phenol potentially prevented shifted the equilibrium position of the bottom reaction in Scheme 3 to the left and enhanced the formation of monosodium salicylate (I) as the main product.

Another plausible effect, as suggested by the theoretical study presented in Scheme 2, is that the reaction is considered reversible in the presence of a solvent due to the solubility of the product (sodium salicylate). The reversibility of the fourth step (k4 in Scheme 2) could result in little or no formation of the desired product, depending on its solubility in the solvent. If the final product, sodium salicylate, precipitates out, this fraction would not participate in the reversible reaction, favouring the forward reaction to make more of the desired product [40]. Giving the increased yield of salicylic acid in these phenol-promoted tests, the presence of phenol could have potentially reduced the solubility of the product in the bulk toluene solvent and enhanced its precipitation. Phenol has a dielectric constant of 12.4 at 30 °C [42], which is considerably higher than that of toluene of 2.4 at 25 °C. Its addition up to molar ratio of 3:1 compared to sodium phenoxide, resulted in a significant increase in the salicylic acid yield. However, when the ratio was increased to 4:1, the salicylic acid yield started to decline but was still more than three times higher than the yield obtained without phenol addition. Increase in the concentration of phenol to 37.5 wt% at the 4:1 molar ratio could have decreased the solubility of CO_2_ in toluene, as well as reducing the probability of its collision with sodium phenoxide or phenolate ion, leading to the observed decrease in eventual salicylic acid yield. The yield of reaction phenol from sodium phenoxide and CO_2_ (Scheme 1) was also calculated as summarised in Figure 3. This shows that the yield of reaction phenol decreased slightly as the molar ratio of phenol increased.

Moreover, a noteworthy observation was made when a 2:1 molar ratio or higher of phenol was introduced into the reaction mixture, where almost equimolar yields of phenol and salicylic acid were obtained. This is consistent with the stochiometric balance shown in Scheme 1.

**Scheme 4:**
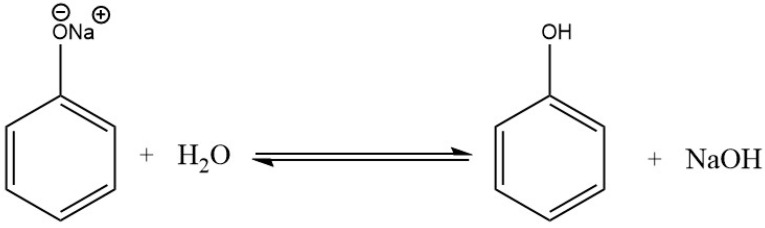
Presence of water due to the hygroscopic nature of the sodium phenoxide which shifts the equilibrium to form phenol.

In addition, sodium phenoxide is very sensitive to moisture and any water presented in the reaction system could hydrolyse phenol as shown in Scheme 4 [27]. The addition of phenol could dilute the presence of water to inhibit this hydrolysis, so that sufficient sodium phenoxide remained in the reaction medium for reaction with CO_2_ to product salicylic acid.

Experimental tests have been conducted to determine if sodium phenoxide would dissociate into phenol and NaOH in toluene in the presence of moisture. In the initial test, toluene was added onto sodium phenoxide immediately after removal from the vacuum oven. The resulting mixture was then placed into the reactor and maintained at 150 °C for 2 h. No phenol was found in the toluene fraction. In a subsequent test, a similar procedure was followed, but excess water (3:1 molar ratio of H_2_O to sodium phenoxide) was introduced to the solids before the addition of toluene. This time 3.5% molar yield of phenol was obtained in the toluene fraction. Although, a relatively small amount of phenol was formed, it would still reduce the amount of sodium phenoxide to react with CO_2_ to form salicylic acid. These tests provided evidence of the negative influence that water could have towards salicylic acid formation, but the possible caging effect of toluene on the sodium phenoxide inhibited the extent of its hydrolysis. This was another benefit of using the toluene solvent.

**Figure A2:**
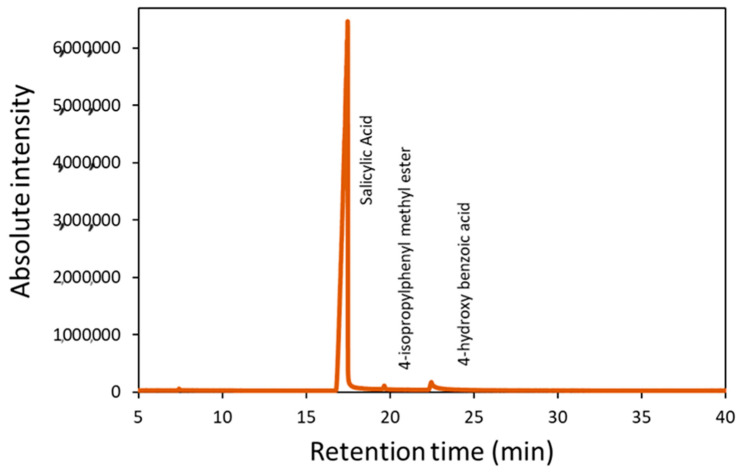
GC/FID chromatogram of acidified solid product showing the high purity/selectivity of salicylic acid.

Formation of side products during the carboxylation of sodium phenoxide in the presence of toluene and at various phenol to sodium phenoxide molar ratio was determined using GC-MS (an example of the GC-MS chromatogram can be found in Appendix A Figure A2).

As shown in Table 2, when the reaction was carried out in the presence of toluene only, significant amounts of bis-phenols were detected. Even though no phenol was added in the initial reaction mixture, the reaction phenol formed during the carboxylation reaction found in the toluene solvent.

The addition of phenol to the reaction mixture in the Kolbe–Schmitt reaction showed significant improvement in the purity of salicylic acid. This improvement suggested that the formation of bis-phenols was likely linked to the conversion of sodium phenoxide. Specifically, at low sodium phenoxide conversions, the remaining unreacted sodium phenoxide transformed into phenol upon acidification. These phenols were subsequently carried over into the final product during the separation step with acetone, which ultimately promoted the generation of bisphenols during the sample work-up process. Since, no bisphenols were detected in the GC-MS chromatogram of the toluene solvent but only in that of the final products, it was considered that bisphenols formed in the last stage, during separation of NaCl from the organics using acetone and dried on a hotplate at 60 °C.

It has been reported that the derivation of bisphenol A was possible by a condensation reaction of phenol and acetone catalysed by HCl at 65 °C [44].

The main side product observed in the presence of added phenol was mainly p-hydroxybenzoic acid. It is noteworthy that the formation of p-hydroxybenzoic acid in the conventional Kolbe–Schmitt reaction has been previously reported, with yields ranging from 1% to 4% [26]. However, in this case much lower yields of this side products were obtained, which was believed to be due to improved mixing during the reaction, preventing agglomeration or hotspots, thus inhibiting the formation of undesired products. Traces of 4-isopropylphenyl methyl ester were also detected, however, this peak was also observed when using a commercial salicylic acid from Thermo Scientific™, Waltham, MA USA, hence it may be a common impurity during salicylic acid synthesis.

#### 2.2.2. Effect of Reaction Time

**Figure 4:**
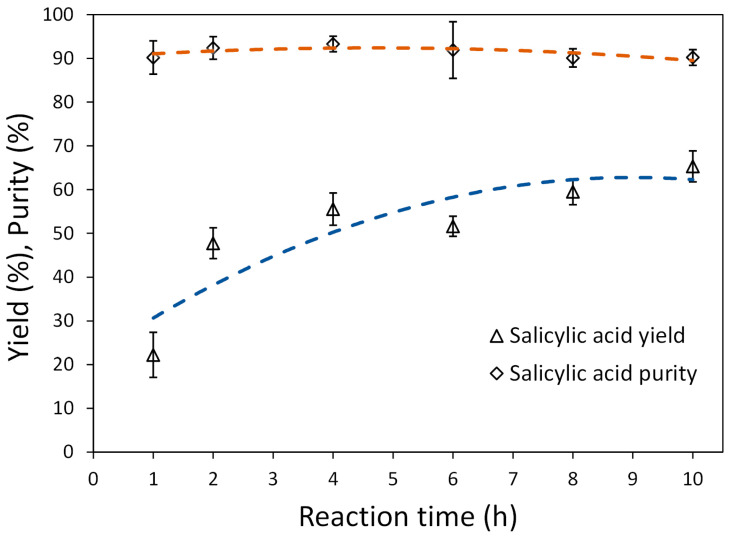
Effect of increase the residence time on the yield of the slurry-based carboxylation. The reaction conditions are as follows: phenol/sodium phenoxide molar ratio (1:1), reaction temperature 150 °C, Pco_2_ = 30 bar, and stirring at 1000 rpm.

To investigate the influence of reaction time on the yields and purity of salicylic acid, all reactions were carried out at 150 °C under a constant partial pressure of CO_2_ (P_CO2_ = 30 bar), 1:1 molar ratio of phenol to sodium phenoxide and stirring speed of 1000 rpm. The results in Figure 4 indicated a linear increase in salicylic acid yields with time, accompanied by a negligible change in the purity of the product. For instance, a residence time of 1 h yielded 22.24% salicylic acid, and the yield more than doubled to 47.73% when the residence time was extended to 2 h. However, a subsequent increase in reaction time beyond 2 h gave less dramatic improvements in the overall yield. The maximum yield of 65.30% was achieved after 10 h of reaction time, while maintaining the purity of salicylic acid at around 90%, with slight increases in yields of side products at longer reaction times.

One of the practical challenges of the industrially applied Kolbe–Schmitt reaction is the long-residence times to enable increased contact of the CO_2_ with dry sodium phenoxide to improve conversion and yields [27]. This prolonged duration is necessary in traditional Kolbe–Schmitt reactions due to the reliance on diffusion for mixing, requiring high CO_2_ pressures to enhance the interaction between the solid–gas reaction mixture. High sodium phenoxide conversions (99%) has been achieved in the standard Kolbe–Schmitt reaction by increasing the reaction times to up to 18 h [27]. In the presence of solvents, the kinetic model from the theoretical study indicated that no product would form even when the residence time was allowed to increase by orders of magnitude due to the reversibility of the final stage of the reaction (Scheme 2). Hence, the reaction could be assumed to be thermodynamically controlled [40].

However, this novel suspension-based novel approach used in this present study, with active mixing to generate adequate amount of turbulence, significantly improved the reaction kinetics. As Figure 4 shows, 44.73% of salicylic acid with a purity of 92% was obtained after two hours. Although, the yield increased by nearly 37% to 65.30% after 10 h of reaction, such as 400% increase in reaction time could not justify its use. In addition to the high of salicylic acid, a 2-h reaction would be more appealing for development into a continuous process. As a result, a reaction time of 2 h was selected as the optimal duration for further optimisation to take advantage of the improved efficiency of this approach.

#### 2.2.3. Effect of CO_2_ Pressure

The influence of CO_2_ partial pressure on the salicylic acid yield and purity was examined in this present suspension-based study by saturating toluene at various reaction pressures ranging from 1 to 50 bar. Generally, high pressures are required to improve the contact between the solid–gas reaction and prevent phenols from volatilising during the conventional Kolbe–Schmitt reaction [27].

**Figure 5:**
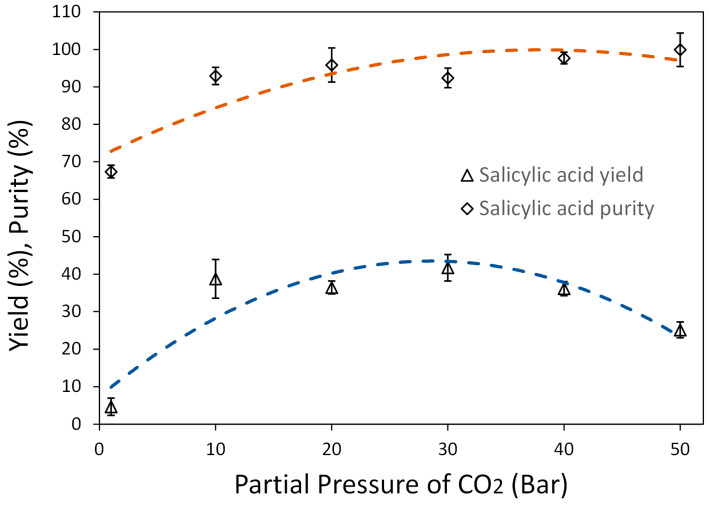
Effect of partial pressure of the CO_2_ on the yield and purity of salicylic acid. The reaction conditions are as follows: phenol/sodium phenoxide molar ratio (1:1), reaction temperature 150 °C, reaction time of 2 h, and stirring at 1000 rpm.

The experiments were carried out at a fixed set of reaction conditions (temperature = 150 °C, reaction time = 2 h, stirring speed of 1000 rpm, and 1:1 molar ratio of phenol to sodium phenoxide). The results obtained showed that carboxylation of sodium phenoxide can take place at partial pressure of CO_2_ as low as P_CO2_ = 1 bar with salicylic yield of 4.64%. The yield significantly increased to 38.73% when the partial pressure of CO_2_ was increased to 10 bar, and maximum yield of salicylic acid (41.71%) was obtained at P_CO2_ = 30 bar. Further increase in P_CO2_ did not improve on the overall yield, for instance at P_CO2_ = 40 bar and P_CO2_ = 50 bar, the yield of salicylic acid was 35.63% and 25.12%, respectively. However, another interesting finding was that the purity of salicylic acid increased with increase of partial pressure of CO_2_, where maximum purity of 99.9% was achieved at P_CO2_ = 50 bar (Figure 5).

The observed trend in the yield of salicylic acid in Figure 5 exhibits a distinctive quadratic pattern, which, to the best of our knowledge, can be attributed to two extreme conditions. Firstly, at low partial pressures of CO_2_, the reaction was hindered by mass transfer limitations, resulting from insufficient availability of CO_2_ in the organic carrier. On the other hand, at higher partial pressures of CO_2_, the vapour molar fraction of toluene decreased $\left(y_{toluene}=y_{CO2}-1\right)$, leading to thermodynamic equilibrium limitations towards the formation of salicylic acid. For instance, the presence of excess CO_2_ (supersaturation) could cause a dramatic change in the dielectric constant of the toluene solvent, thereby influencing the solubility of both sodium phenoxide reactant and the sodium salicylate products in toluene. The reduced solubility of sodium phenoxide would make it unavailable for reaction with CO_2_, whereas the increased solubility of the sodium salicylate product could promote its reversible degradation. Between these two, the more plausible to cause a reduction in salicylic acid yield would be the reduced availability of sodium phenoxide.

Figure 5 shows that the purity of the salicylic acid produced generally increased with increased CO_2_ pressure. The possible reduced solubility of sodium phenoxide would reduce its concentration in the reaction medium and this could prevent concentration-based side reactions. In addition, the increasing purity could also be supported by the enhanced precipitation rate of the sodium salicylate product once formed, thereby avoiding being subjected to further reactions within the solvent.

#### 2.2.4. Effect of Reaction Temperature

**Figure 6:**
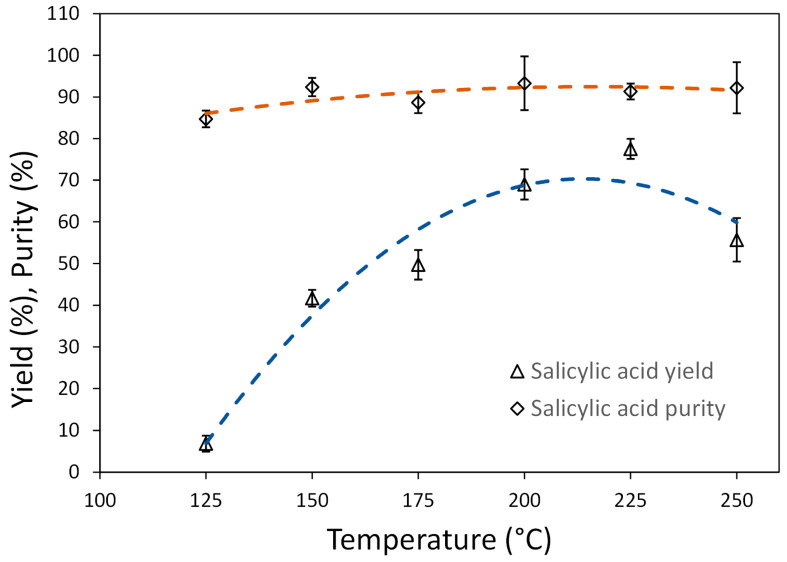
Effect of temperature on the yield and purity of the slurry-based carboxylation (reaction conditions are as follows, phenol/sodium phenoxide molar ratio (1:1), reaction time of 2 h, Pco_2_ = 30 bar and stirring, 1000 rpm).

The effect of reaction temperature on the yields and purity of salicylic acid from the suspension-based reaction of sodium phenoxide and CO_2_ was investigated between a temperature range of 125 °C and 250 °C based on 25 °C increments. While the rate of reaction was expected to increase linearly with the rise in temperature since it was thought that the reaction was no longer thermodynamically restricted at moderate pressures (P_CO2_ = 30 bar) (Figure 5). Figure 6 shows that the experimental results were in good accord with the hypothesis of the reaction being kinetically limited at moderate pressures.

**Scheme 5:**
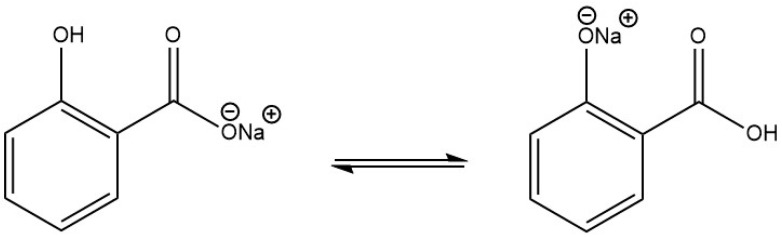
Equilibrium of the phenolate form and carboxylate form of sodium salicylate.

**Scheme 6:**
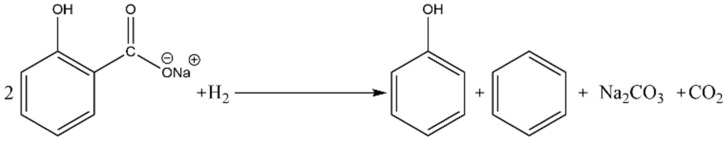
Composition of products confirmed by the TGA-FTIR study of the thermal decomposition of sodium salicylate [43].

For instance, at 100 °C the reaction did not yield any salicylic acid, but for a further increase in temperature to 125 °C, the yield of salicylic acid rose to 6.84%. A significant increase in the yield of salicylic acid (41.71%) was observed at 150 °C, which is the preferred condition in the conventional Kolbe–Schmitt reaction [7]. The maximum yield of salicylic acid (77.52%) was achieved at 225 °C. When the temperature was raised to 250 °C, the yield of salicylic acid suddenly dropped to 55.09%. It was reported that sodium salicylate has a great propensity to undergo thermal decomposition at ~240 °C due to equilibrium between the carboxylate and the phenolate salts (Scheme 5). Hence the promotion of the decarboxylation of the phenolate salt at higher temperatures would permanently reverse the reaction to make sodium phenoxide and CO_2_ as shown in Scheme 6 [43].

Additionally, a TGA-FTIR study conducted by Zhang [43], showed that benzene and phenol were produced during the decomposition of sodium salicylate (Scheme 6). Hence, the poor reduced thermal stability of the sodium salicylate salt at 250 °C could explain the observed decrease in yield of salicylic acid at this temperature.

In the Marrase-modified method, increase in temperature favoured formation of 1,4-benzenedicarboxylic acid, accounting for 36% of the total product composition [26]. In this present study, increase in temperature did not have much impact on the purity of the final product from the suspension-based carboxylation. The average purity obtained was ~90%, except when the reaction was carried out at 125 °C where the purity was 84.72%.

#### 2.2.5. Effects of Stirring Speeds Salicylic Acid Yields under Optimised Reaction Conditions

**Figure 7:**
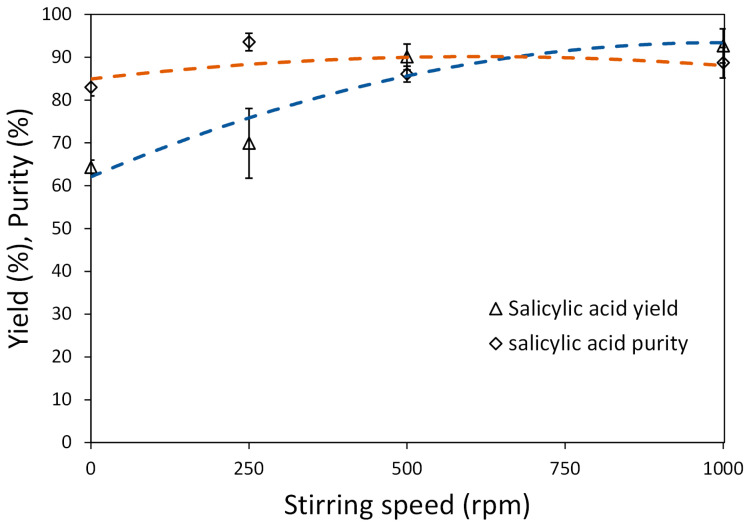
Effect of stirring speed on the yield and purity of salicylic acid from slurry-based carboxylation. Reaction conditions: phenol/sodium phenoxide molar ratio = 2:1, reaction time = 2 h, reaction temperature = 225 °C, and Pco_2_ = 30 bar.

Through careful consideration of various factors could help to identify a set of optimal conditions for the slurry-based Kolbe–Schmitt reaction. Using 2 moles of phenol ensured a favourable yield the desired product, while a reaction time of 2 h struck a balance between yield and productivity. A partial pressure of 30 bar of CO_2_ improved both yield and purity, while a reaction temperature of 225 °C maximised the yield of sodium salicylate (without being too hot to cause thermal decomposition). To elucidate the impact of both passive and active mixing on the suspension-based Kolbe–Schmitt reaction, a series of experiments with stirring speeds ranging from 0 to 1000 rpm (rpm) was carried out under the optimised conditions. The results are summarised in Figure 7.

Giving that the reaction conditions were fixed, the yield of salicylic acid within 2 h of reaction from the different experiments would give an indication of the rate of reaction leading to its formation. The results showed that the rate of reaction improved with increasing stirring speed. Indeed, the yield of salicylic acid increased proportionally to increased rate of mixing as the stirring speed increased, showing that the reaction was essentially diffusion controlled. For instance, at 0 rpm (no forced mixing), the salicylic acid yield was 64.28%. Evidently from Figure 7, as the stirring rate gradually increased, the yield of salicylic acid exhibited a consistent upward trend up until 500 rpm. However, only marginal increases in salicylic acid yield were observed beyond 500 rpm. The highest yield of salicylic acid obtained was 92.68% at 1000 rpm, whereas it was 90.08% at 500 rpm. While the yield of salicylic acid improved with mixing, the purity of the product was largely unaffected. Although, the synergistic effect of stirring and optimised conditions helped to achieve the impressive yield with high purity of salicylic acid, it is anticipated that making the particle size of sodium phenoxide smaller may further increase the reaction rate.

### 2.3. Comparison of Lab-Synthesised Salicylic Acid vs. Commercial Analogue

**Figure 8:**
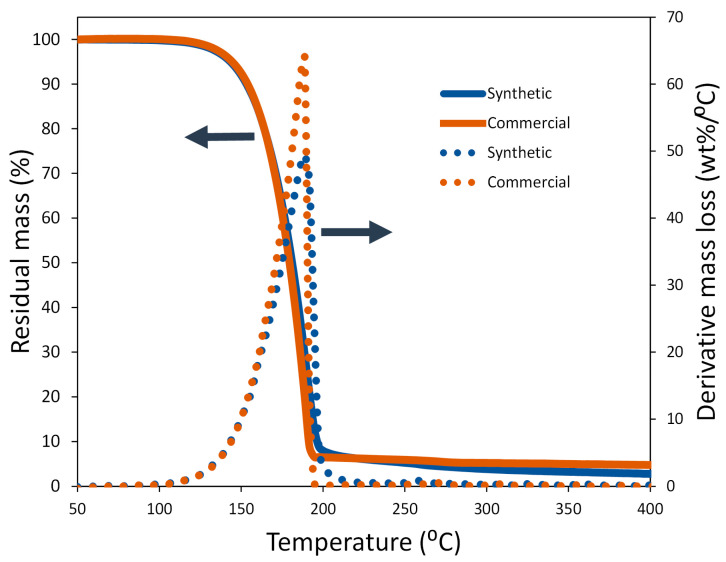
TGA (left y-axis) and DTG (right y-axis) curves for commercial and the one produced in the lab salicylic acid.

To consolidate the accuracy of the results obtained via GC-FID, TGA was conducted on commercially available salicylic acid (+99%) and the one produced in this present work. For this test, the salicylic acid produced under reaction conditions of phenol/sodium phenoxide molar ratio (2:1), reaction temperature 225 °C, reaction time of 2 h, stirring, 1000 rpm and Pco_2_ = 30 bar was used, as it gave the highest yield with purity of 90.1%. The boiling point of salicylic acid is 211 °C [45]. The TGA results in Figure 8 shows that the residual mass loss (%) of both commercial and the lab produced salicylic acid almost dropped to 10% between the range of 120 and 210 °C. Figure 8 also shows that the maximum mass loss of the commercial salicylic acid occurred at a peak temperature (Tmax) of 180 °C, whereas for the one produced in this present study, the Tmax occurred at 185 °C (Figure 8). The slight difference is believed to be due to presence of impurities. For this particular salicylic acid, the purity obtained from the GC-FID analysis was in good accord with the TGA results.

**Figure 9:**
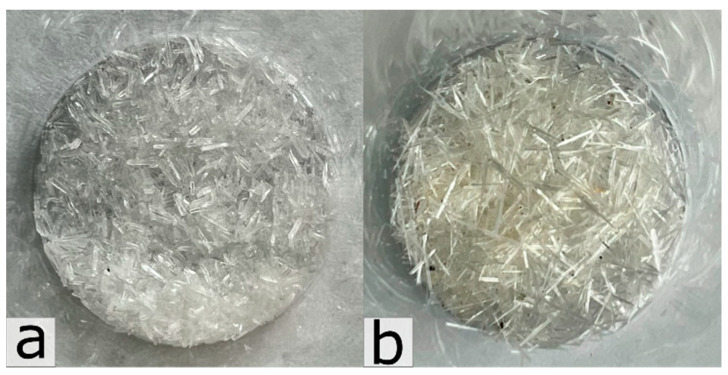
A comparison of salicylic acid samples. (**a**) Salicylic acid obtained commercially. (**b**) Salicylic acid synthesised in this study.

The photos of salicylic acid produced in the lab and commercial acquired are shown in Figure 9. While the slight discolouration of the salicylic acid from this present work is noticeable in Figure 9b, this was still an impressive result considering that the isolation method used was developed in-house using simple techniques such as pH changes, dissolution and recrystallisation. More laborious isolation methods have been described in the literature, including dissolution in water, treatment with activated charcoal, and sublimation of salicylic acid [25]. However, further refining of the final product would be development for application at a larger scale production to achieved higher purity of salicylic acid.

## 3. Materials and Methods

### 3.1. Materials

The chemicals that were used for this slurry-based Kolbe–Schmitt reaction included phenol (C_6_H_5_OH, 94.11 g/mol, 99% extra pure; Thermo Scientific™, Waltham, MA USA), sodium hydroxide (NaOH, 39.997 g/mol, ≥98%, Honeywell, Skimped Hill Ln, UK), sodium phenoxide (C_6_H_5_ONa, 116 g/mol, 98%; Thermo Scientific™), salicylic acid (C_6_H_4_(OH)COOH, 138 g/mol, +99%; Thermo Scientific™), de-ionised water (Q-pod system, 0.22 µm, carbon dioxide (CO_2_, 44.01 g/mol, CP Grade; BOC), toluene (C_7_H_8_, 92.13 g/mol, 99.5%, ACS reagent, Thermo Scientific™), and acetone ((CH_3_)_2_CO, 58.08 g/mol, 99.5%, HPLC Grade, Thermo Scientific™).

### 3.2. Preparation of Sodium Phenoxide

Sodium phenoxide was synthesised according to Kolbe’s recommended method, which was first reported in 1860 [25,27] and is now practically used at industrial scale. In this present study, sodium phenoxide was prepared by dissolving 40 g of phenol in slightly less than an equimolar 50 wt% sodium hydroxide solution in a glass liner that fits into a 450 mL stainless still reactor vessel. After loading, the glass liner was placed inside the main 450 mL 4575A fixed head bench top Parr reactor vessel, consisting of a vertical closed autoclave equipped with a stirrer. The reactor was heated in a jacket to 130 °C for 4 h at a stirring rate of 50 rpm.

Subsequently, the reactor was cooled down to 40 °C and the reddish solution containing sodium phenoxide solution was transferred into a separate beaker. The beaker was placed inside a vacuum oven at 60 °C to dry for several hours until a white-reddish solid sodium phenoxide was obtained. The dry sodium phenoxide solid was removed from the beaker, crushed, sieved to particle size of 125–250 µm and stored in an air-tight container prior to use.

### 3.3. Thermal Gravimetric Analysis (TGA)

The formation and purity of the prepared sodium phenoxide was determined using METTLER TOLEDO TGA/DSC 3+ thermogravimetric instrument. About 4 mg samples were loaded in a 70 µL alumina crucible and then heated from 50 to 900 °C at a rate of 10 °C/min in nitrogen (60 mL/min). The thermogram of the prepared sodium phenoxide was compared with that of the commercial sample. The same TGA instrument was also used to determine the purity of salicylic acid in the solid product obtained from the reaction.

### 3.4. Suspension-Based Carboxylation Reaction

**Figure 10:**
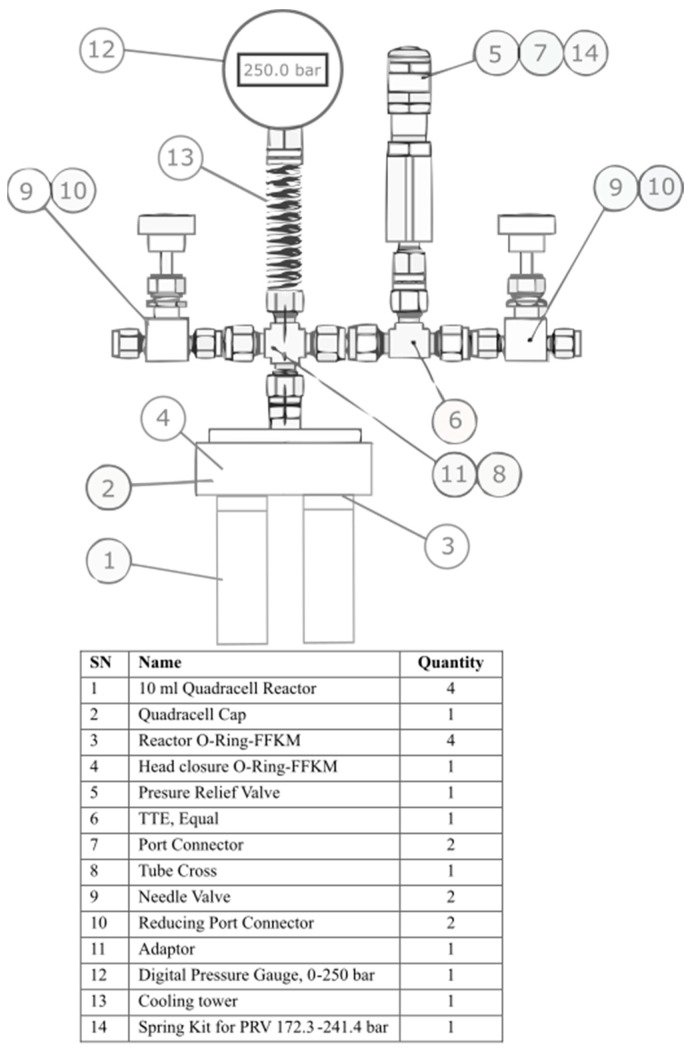
Asynt quadracell reactor with integrated cooling tower and a digital pressure gauge.

The suspension-based carboxylation reactions were carried out in a set of 4 × 10 mL Quadracell reactors supplied by Asynt, Isleham, Cambridgeshire, United Kingdom. In each cell, a known amount of prepared sodium phenoxide (0.3 g) was weighed. Then, 6 mL of toluene with various phenol concentrations was mixed with the sodium phenoxide, forming a suspension. Afterward, all four reactor cells, each containing a magnetic stirrer, were sealed onto the main reactor cap and purged with CO_2_ to remove the air. The reactor system was pressurised until it reached to the designated operating pressure (1–50 bar). This pressure was achieved by employing a two-stage piston cylinder regulator (GASARC, Tech Master GPT420 Series). The regulator valve was used to set the maximum delivery of the desired pressure, and a digital pressure transducer was employed to monitor the pressure inside the reactor until it reached saturation, indicating that no further dissolution of CO_2_ in the organic carrier was occurring. The reactors were heated on the designated hotplate to the desired temperature (100–250 °C) at a heating rate of 10 °C/min and a stirring rate of 0–1000 rpm. The reaction mixture was held for a specified duration (1–10 h), before being allowed to cool down to room temperature. The reaction temperatures and stirring speed were regulated by an Asynt ADS-HP-NT magnetic stirrer hotplate and the pressure was displayed using a digital pressure gauge integrated with a cooling tower, see Figure 10. 

### 3.5. Post-Reaction Treatment

After the reaction, the gases were collected in a 1 L Tedlar sampling bag for offline analysis. Vacuum filtration was then used to remove the solid phase from the organic carrier. After being vacuum dried, the solid phase was treated by acid precipitation method using concentrated HCl (12 M). The acidification process helped to decompose any carbonates formed, while the phenolic salts of sodium (e.g., sodium phenolate and sodium salicylates) were converted to phenol and solid salicylic acids, respectively. The reactions of HCl and the sodium ion present also led to the formation of NaCl. The formation of NaCl would also include the reaction between HCl and any carbonates of sodium (Na_2_CO_3_/NaHCO_3_) formed during the reaction. The solid products (precipitates) containing mainly salicylic acids and NaCl were dried at 105 °C for 2 h. Acetone was used to dissolve the organics, and NaCl was separated once again by vacuum. In the last stage, the acetone solvent was driven off by gentle heating on a hotplate at 60 °C, until crystals started to form.

### 3.6. Gas-Phase Products

**Figure A3:**
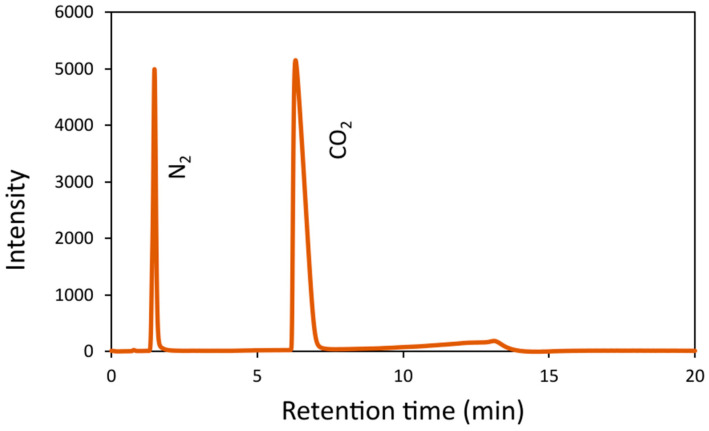
GC/TCD/FID chromatogram showing (inert diluent) and CO_2_ as only gas products after reaction.

The gas collected in the Tedlar bag was analysed using a Shimadzu GC-2014 gas chromatograph (Kyoto, Japan). The analytical conditions used for this instrument have been reported in previous publication by the research group [46]. Several analyses of the gas samples were carried out and the chromatograms only showed the presence unreacted CO_2,_ and no other hydrocarbon gases species were formed (Appendix A Figure A3).

### 3.7. Gas Chromatography–Mass Spectrometry (GC–MS) for Qualitative Analysis of Salicylic Acid and Phenol

A new protocol for identification of salicylic acid and the solvent carrier (phenol in toluene) was achieved using Shimadzu gas chromatography–mass spectrometer (GC–MS—QP2010 SE) equipped with a SH-Rxi™-5ms capillary Colum (diphenyl dimethyl polysiloxane, 30 m, 0.25 mm id, 0.25 µm) using a 1:50 split ratio operating at 50 °C for 2 min, then heated at 5 °C/min up to 300 °C. A quadrupole mass detector was used, with electron ionisation relative to the tuning result, operating in the range of 20–250 *m*/*z*.

### 3.8. Chromatography–Flame Ionisation Detection (GC–FID) for Quantification of Salicylic Acid and Phenol

**Figure A4:**
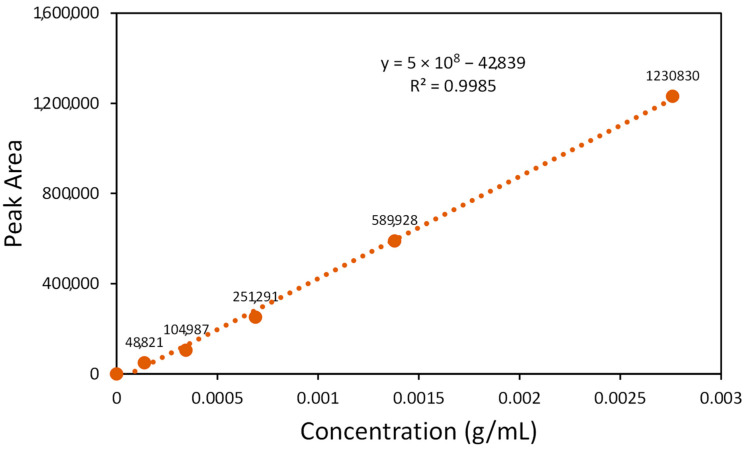
GC/FID calibration data of salicylic acid.

A gas chromatography–flame ionization detection (GC–FID) method was used for direct quantitative analysis of salicylic acid using external standard method [47,48]. The chromatography was performed by using a gas chromatograph (SHIMADZU, GC-2010 Plus) equipped with an FID. An Rtx^®^-5MS fused silica column (diphenyl dimethyl polysiloxane, 30 m, 0.25 mm id, 0.25 µm) was used. The carrier gas (nitrogen) flow rate was set to 0.90 mL/min. Injector and detector temperatures were 300 °C and 280 °C, respectively. For analysis, 1 µL of salicylic acid was injected with a split ratio of 1:20. The oven temperature program was set at 40 °C for 5 min, then ramped to 280 °C at 10 °C/min and held for 10 min. The calibration curve for the external method was constructed by injecting known concentrations of salicylic acid solutions in acetone. The generated standard curve correlated various peak areas to the known mass of salicylic acid. The purity of salicylic acid was calculated based on the plotted calibration curve using the following formula:

(1) $$A_{i}=m_{i}M_{SA}+c$$

where $A_{i}$ is the chromatographic area, $m_{i}$ is the slope of the curve, $M_{SA}$ is the mass of salicylic acid in g, and c is the y-intercept constant. The equation was rearranged for $M_{SA}$ and chromatographic peak area of the unknown salicylic acid was obtained from GC–FID. The accuracy of the measurement was also determined by treating a known mass of salicylic acid (0.0069 g diluted in 10 mL of acetone) as an unknown sample, resulting in +99% accuracy. (An example of GC–FID calibration data of salicylic acid can be found in Appendix A Figure A4)

The yield and purity of salicylic acid was calculated using a standard formula as shown below [49]:

(2) $$Yield\left(\%\right)=\frac{Obtained\;molar\;Yield}{Theoretical\;molar\;Yield}\times 100$$

Based on reaction presented in Scheme 1

(3) $$Purity\left(\%\right)=\frac{M_{SA}\;}{\sum M_{TSP}\;}\times 100$$

where $M_{SA}$ is the mass of salicylic acid from the chromatographic peak area in g. $\sum M_{TSP}$ is the total mass of the solid product (g) obtained after the extraction and drying stage.

A similar approach for the quantification of phenols was used, where a calibration curve for the external method was constructed by injecting known concentrations of phenol in toluene instead.

The experiments were repeated 2–3 times, and the error margins were determined as the standard deviation of the yield of salicylic acid and the purity of the solids based on the GC–FID results. The results of error analyses are presented as error bars in the respective figures.

The overall methodology of the experiments, from the preparation of the sodium phenoxide up until the isolation and quantification of salicylic acid is summarised in (Figure 1). It is worth highlighting that the phenol produced in the organic carrier can be readily separated through simple distillation, due to the difference in boiling points of toluene (110 °C) and phenol (182 °C). Alternatively, a sustainable separation method involves utilising water as a green solvent to extract phenols from the organic carrier, toluene. This can be followed by a straightforward decantation process at ambient temperature, potentially offering a more cost-effective solution than distillation. Subsequently, the water–phenol mixture obtained can be utilised as a feedstock for sodium phenoxide production.

## 4. Conclusions

The experimental results of this study showed that the carboxylation of sodium phenoxide solely in the presence of toluene yielded 18.70% salicylic acid under the reaction conditions of 150 °C, CO_2_ pressure of 30 bar, and a reaction time of 2 h. However, upon the addition of phenol as a promoter at the same mole amount of sodium phenoxide under identical reaction conditions, the salicylic acid yield notably increased to 45.23%. Upon further optimisation of the reaction parameters, an outstanding salicylic acid yield of 92.68% was achieved under the optimal conditions of a phenol/phenoxide molar ratio of 2:1 in toluene, a reaction temperature of 225 °C, a CO_2_ pressure of 30 bar, a reaction time of 2 h, and stirring at 1000 rpm.

Investigation into the effects of phenol addition revealed intriguing insights into the thermodynamic equilibrium of the carboxylation reaction. Phenol inhibited the formation of undesired side products, thereby enhancing the overall yield of salicylic acid. The equimolar yield of phenol and salicylic acid at specific molar ratios highlighted the stoichiometric balance critical for maximizing product formation.

Additionally, the experiments in this study elucidated the impact of moisture on the reaction equilibrium of sodium phenoxide, with phenol addition proving effective in mitigating the hydrolysis of sodium phenoxide. This finding underscored the importance of reaction conditions and additives in controlling side reactions and maximizing the desired product yields.

This outstanding outcome represents a breakthrough to carry out the Kolbe–Schmitt carboxylation reaction in a suspension in terms of the significantly shortened reaction time, the reduced CO_2_ pressure, a much higher yield of salicylic acid, and very limited yields of undesired side products. Compared to the conventional Kolbe–Schmitt reaction, the suspension-based carboxylation approach could open the opportunity for the adoption of continuous flow reactor technology for industrial-scale carboxylation reactions. Continuous flow reactors offer several advantages, including improved heat transfer, energy efficiency, and scalability, contributing to safer and more economic CO_2_ utilisation and, probably, storage.

Obtaining a nearly 93% yield of salicylic acid at 225 °C with 30 bar CO_2_ pressure for 2 h would provide considerable energy savings compared to one of the best reported yields in the literature of 79% after 8 h at 125 °C with up to 138 bar of CO_2_. The detailed energy consumption of this novel process will be considered as part of future studies. The temperature range used in this present work is easily affordable with renewable electricity from wind and solar, which makes the process even more promising.

## Figures and Tables

**Table 1:** The Kolbe–Schmitt type reaction conducted by other researchers using solvents with various dielectric constant [27,38].

Solvent	Dielectric Constant	Temperature (°C)	Pressure (bar)	* SA Yield (wt%)
Methanol	31.2	140	22	0
Ethanol	25.8	140	6	0
1-Butanol	19.2	155	10	7.5
Glycol	41.2	140	6	0
Glycerol	56.2	170	10	0
Xylene	2.6	138	1	33.5
Di-isobutyl ketone	9.9	150	1	18.9

* SA = Salicylic acid.

**Table 2:** Normalised GC-MS peak area (%) at various phenol to sodium phenoxide molar ratios.

Mole Ratio (Phenol/Sodium Phenoxide)					
	0:1	1:1	2:1	3:1	4:1
Compounds	Peak Area (%)				
Salicylic acid	48.76	99.13	98.60	98.33	97.70
Phenol	0.00	0.00	0.91	0.07	0.08
4-isopropylphenyl methyl ester	3.20	0.35	0.29	0.21	0.24
P-hydroxybenzoic acid	0.25	0.51	0.19	1.39	1.90
4-(1,1-Dimethylpropyl)phenol	2.68	0.00	0.00	0.00	0.00
2,4′-Bisphenol A	2.70	0.00	0.00	0.00	0.00
4,4′-Bisphenol A	38.13	0.00	0.00	0.00	0.00
1,4-Benzenedicarboxylic acid	0.31	0.00	0.00	0.00	0.08
Phthalic anhydride	3.98	0.00	0.00	0.00	0.00

## Data Availability

Most research data have been included in this paper. Additional data are available upon request.
